# Evaluating the Probiotic Potential of Lactic Acid Bacteria Implicated in Natural Fermentation of Table Olives, cv. *Cobrançosa*

**DOI:** 10.3390/molecules28083285

**Published:** 2023-04-07

**Authors:** Joana Coimbra-Gomes, Patrícia J. M. Reis, Tânia G. Tavares, Miguel A. Faria, F. Xavier Malcata, Angela C. Macedo

**Affiliations:** 1 LEPABE—Laboratory for Process Engineering, Environment, Biotechnology and Energy, Faculty of Engineering, University of Porto, Rua Dr. Roberto Frias, 4200-465 Porto, Portugal; joanacoimbragomes@gmail.com (J.C.-G.); pmreis@fe.up.pt (P.J.M.R.); tsgtavares@fe.up.pt (T.G.T.); amalcata@umaia.pt (A.C.M.); 2 ALiCE—Associate Laboratory in Chemical Engineering, Faculty of Engineering, University of Porto, Rua Dr. Roberto Frias, 4200-465 Porto, Portugal; 3 LAQV/REQUIMTE, Laboratory of Food Science and Hydrology/Rede de Química e Tecnologia, Department of Chemical Sciences, Faculty of Pharmacy, University of Porto, Rua de Jorge Viterbo Ferreira 228, 4050-313 Porto, Portugal; mfaria@ffup.up.pt; 4 UNICES-UMAIA—Research Unit in Management Sciences and Sustainability, University of Maia, Av. Carlos Oliveira Campos, 4475-690 Maia, Portugal

**Keywords:** *Lactiplantibacillus* strains, functional properties, Caco-2 cell adhesion, autoaggregation, antioxidant activity, cholesterol assimilation

## Abstract

The probiotic features of *Lactiplantibacillus* (L.) *pentosus* and *L. paraplantarum* strains, endogenous in *Cobrançosa* table olives from northeast Portugal, were assessed in terms of functional properties and health benefits. Fourteen lactic acid bacteria strains were compared with *Lacticaseibacillus casei* from a commercial brand of probiotic yoghurt and *L. pentosus* B281 from Greek probiotic table olives, in attempts to select strains with higher probiotic performances than those references. For functional properties, the i53 and i106 strains, respectively, exhibited: 22.2 ± 2.2% and 23.0 ± 2.2% for Caco-2 cell adhesion capacity; 21.6 ± 7.8% and 21.5 ± 1.4% for hydrophobicity; 93.0 ± 3.0% and 88.5 ± 4.5% for autoaggregation ability by 24 h of incubation; and ability to co-aggregate with selected pathogens—from 29 to 40% to Gram+ (e.g., *Staphylococcus aureus* ATCC 25923 and *Enterococcus faecalis* ATCC 29212); and from 16 to 44% for Gram− (e.g., *Escherichia coli* ATCC 25922 and *Salmonella enteritidis* ATCC 25928). The strains proved to be resistant (i.e., halo zone ≤14 mm) to some antibiotics (e.g., vancomycin, ofloxacin, and streptomycin), but susceptible (i.e., halo zone ≥ 20 mm) to others (e.g., ampicillin and cephalothin). The strains exhibited health-beneficial enzymatic activity (such as acid phosphatase and naphthol-AS-BI-phosphohydrolase), but not health-harmful enzymatic activity (such as β-glucuronidase and N-acetyl-β-glucosaminidase). Additionally, the antioxidant activity and cholesterol assimilation features, respectively, of the strains were 19.6 ± 2.8% and 77.5 ± 0.5% for i53, and 19.6 ± 1.8% and 72.2 ± 0.9% for i106. This study indicated that the addition of *L. pentosus* strains i53 and/or i106 to *Cobrançosa* table olives is likely to enhance the added value of the final product, in view of the associated potential benefits upon human health.

## 1. Introduction

The food industry has grown recently in response to consumer demand for higher quality food and food bearing positive effects upon human health. The latter belongs to the so-called family of “functional foods”—defined as matrices with the addition of nutraceutical ingredients, or subjected to minimum food processing to enhance bioactivity, but always providing health benefits beyond the provision of essential nutrients if regularly consumed as part of a balanced diet [1]. One example is food containing probiotics—i.e., viable microorganisms that, if ingested to appropriate levels, can exert several beneficial effects upon human health [2]. This is a dynamic field of endeavour [3], encompassing studies of probiotic potential of strains, ranging from native strains in traditional cheeses [4,5] to fish intestinal microbiota [6]. As per the high demand for probiotic products of non-dairy origin, researchers have explored survival and stability of probiotic strains in fruit, vegetable, cereal, meat, and soy food matrices. For instance, use of meat as a vehicle helps probiotic bacteria face adverse processing conditions due to its high fat content; use of fruits as vectors takes advantage of their unique physiology as effective environmental protectants of probiotic microorganisms—namely in the reduction in exposure to severe gastrointestinal conditions—further enhancing proliferation due to their high sugar content. It is also important to note that probiotic stability is highly dependent on the microorganism strain vs. processing parameters—e.g., the low pH of fruit juices favours survival of lactobacilli when compared to bifidobacteria, while the fibre present in cereals promotes growth of bifidobacteria. Table olives represent a unique form of delivery, integrating high fibre content with high fat content—thus conveying a hybrid medium of meaty and fruity features [7]. They are one of the oldest fermented foods and are an integral component in the Mediterranean diet.

Despite bearing a regional economic significance, table olive fermentation is still craft-based and empirically driven by its autochthonous microbiota. As an essentially spontaneous process, the quality of the final product hinges critically on various intrinsic and extrinsic factors, hence unpredictable and variable quality are commonplace [8,9]. Although a few nonconventional microorganisms have shown promising features toward food fermentation, comprehensive research efforts are still needed. The most important challenges concerning probiotic properties of microorganisms implicated in natural fermentation are safety issues, high cost, lack of process standardization, satisfaction of regulatory frameworks, and lack of detailed knowledge of their functionality in actual food products [10,11].

Among the multitude of microorganisms found during olive fermentation, Lactic Acid Bacteria (LAB) are the main bacteria responsible for brine acidification; this is due to their lactic acid production which leads to pH decrease, thus contributing to microbiological stability in the final product and extended shelf life. Oftentimes, LAB are candidates as potential probiotic microorganisms for withstanding the adverse conditions prevailing in the human gastrointestinal tract, prior to colonizing the gut [8,12]. Certain probiotic LAB species are typically associated with cultivar, e.g., *Lactobacillus parafarraginis* with Manzanilla [13], or *L. paraplantarum* with *Kalamata* and *Conservolea* [14], or *Tonda di Cagliari* [15]. To date, more than 40 species belonging to nine genera of LAB have been identified [16]; the genus most often cited is *Lactobacillus*, with *L. plantarum* and *L. pentosus* as the most frequent species—irrespective of country, processing method, or olive type. Examples of such probiotic LAB strains include those isolated from olives in Cyprus, Greece, Italy, Morocco, Portugal, and Spain [16,17]. In Portugal specifically, cultivars *Galega* [18] and *Negrinha do Freixo* [19,20] have already been studied in this regard, although the latter currently holds a Protected Designation of Origin [21]; recent interest has arisen in cultivar *Cobrançosa*—originated in Mirandela in northern Portugal, and widely known for its unique sensory features and long shelf life. Several relevant probiotic traits appear to be strain-specific, thus compromising straightforward extrapolation (even within the same species) from previous studies [22,23]; this realization helps justify the timeliness and relevance of this study.

*Cobrançosa* table olives undergo a two-stage processing: (i) sweetening stage—table olives are washed periodically with spring water of different proportions, and kept thereafter in water or brine with low salt content (up to 4%) for 4–6 months; and (ii) salting stage—salt is gradually added to the brine, up to 7–10% (*w*/*w*), but water/brine is no longer changed until the product is ready for market (up to 11 months) [24].

Overall, the main goal of this study was to evaluate the probiotic potential of 14 selected *Lactoplantibacillus* strains isolated from *Cobrançosa* cultivar. This was achieved by running tests that were split into two categories: functional properties—related to (expected) strain performance along the human gastrointestinal tract, thus critical to ascertain their chance of exerting a probiotic function once in the colon; and health benefits—associated with specific bioactivities exhibited in vitro, thus likely to also be present in vivo in the colon. The study of the probiotic potential of LAB will eventually allow for development of a starter or adjunct culture with commercial expression, specifically adapted to fermentation of *Cobrançosa* olives. By doing so, a final product of higher quality is anticipated—bearing improved functional properties in addition to their pre-existing sensory and nutritional features. Therefore, probiotic *Cobrançosa* table olives will likely reach a premium price in the modern food market, thus contributing to sustainable socio-economic and industrial development of the northern region of Portugal.

## 2. Results

### 2.1. Functional Properties

The results pertaining to functional properties, such as adhesion to Caco-2 cells, hydrophobicity, autoaggregation, and co-aggregation, for the 14 LAB native strains (belonging to genus *Lactiplantibacillus* (*L.*), and species *L. paraplantarum* and *L. pentosus,* previously collected from *Cobrançosa* table olive brines [24]) (see Table 1), additionally of two probiotic reference strains (from animal and plant sources), are tabulated in Table 2 and Table 3.

#### 2.1.1. Caco-2 Cell Adhesion

Statistical differences between LAB cultures were found at the 5% level of significance (see ANOVA *p*-value in Table 2). For instance, strain i106 exhibited the highest value of Caco-2 cell adhesion (denoted by superscript ^a^), which was statistically similar to that of strain i53 (also denoted by superscript ^a^), but statistically different from the others (accordingly lacking superscript ^a^). Comparatively, strain i24 exhibited the lowest value of Caco-2 cell adhesion (denoted by superscript ^i^), which was statistically similar to that of strains i21, i26, i32, i34, and i108 (also denoted by superscript ^i^), but statistically different from the others (accordingly lacking superscript ^i^). Upon comparison of our results of adhesion to those encompassing the strain isolated from Greek table olives, it was concluded that strains i106, i53, and i101, exhibit similar performance—with scores of 23, 22, and 19%, respectively. Conversely, strains i21, i24, i32, i34, and i108, exhibited adhesion significantly lower than the yoghurt probiotic strain, viz. 6.2, 5.3, 6.0, 6.1, and 4.8%, respectively. The other strains displayed significantly higher adhesion than that of commercial probiotic yoghurt, but lower than that of Greek olives.

#### 2.1.2. Hydrophobicity

Statistical differences between LAB cultures were found at the 5% level of significance (see ANOVA *p*-value in Table 2). Both reference strains performed similarly with regard to hydrophobicity—27 and 32% for yoghurt and table olives, respectively; most tested strains exhibited, in general, behaviours similar to those of the reference strains. Strain i21 showed significantly higher hydrophobicity (56%, denoted by superscript ^a^), followed by strain i32 (41%, denoted by superscript ^b^); whereas lower hydrophobicity was recorded for four strains—i19 (16%), i101 (19%), i112 (19%), and i108 (21%) (all denoted by superscript ^de^).

#### 2.1.3. Autoaggregation

At the 5% level of significance (see ANOVA *p*-values in Table 2), no statistical differences between LAB cultures were apparent by 5 h, but the reverse occurred by 24 h. For instance, strain i53 exhibited the highest value of autoaggregation value by 24 h of incubation (ca. 93%, denoted by superscript ^a^), whereas i22 exhibited 59% as the lowest value of autoaggregation (denoted by superscript ^d^); the other strains exhibited autoaggregation performance statistically similar to the commercial yoghurt strain (denoted by superscript ^abcd^).

#### 2.1.4. Co-Aggregation with Pathogens

Of all combinations of LAB and pathogenic strains assessed, co-aggregation ranged from 16% pertaining to strain i53 and *S. enteritidis*, to 46% pertaining to strain i26 and *C. albicans* (see Table 3). However, no significant differences were perceived between our strains and the reference strains.

#### 2.1.5. Relationship between Functional Properties

**Figure 1:**
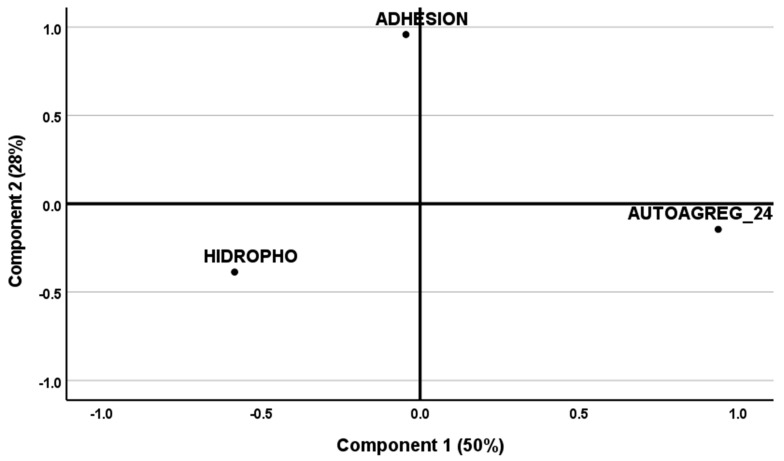
Plot of communalities from component extraction method, pertaining to probiotic characteristics associated with gastrointestinal tract.

**Figure 2:**
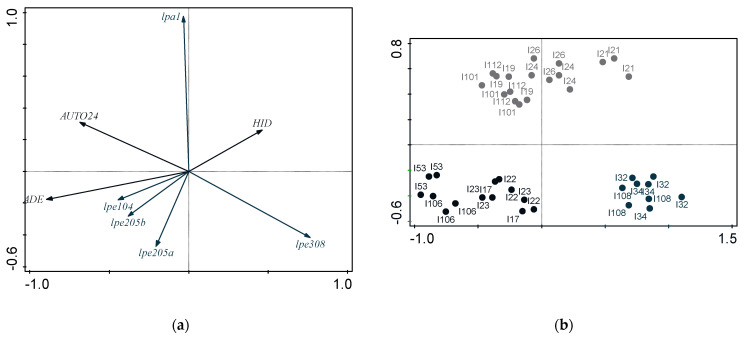
(**a**) Plot of loadings formed by first two principal components from Principal Component Analysis, pertaining to probiotic characteristics associated with gastrointestinal tract and RAPD profile; and (**b**) plot of scores encompassing strains isolated from *Cobrançosa* table olives.

**Figure 3:**
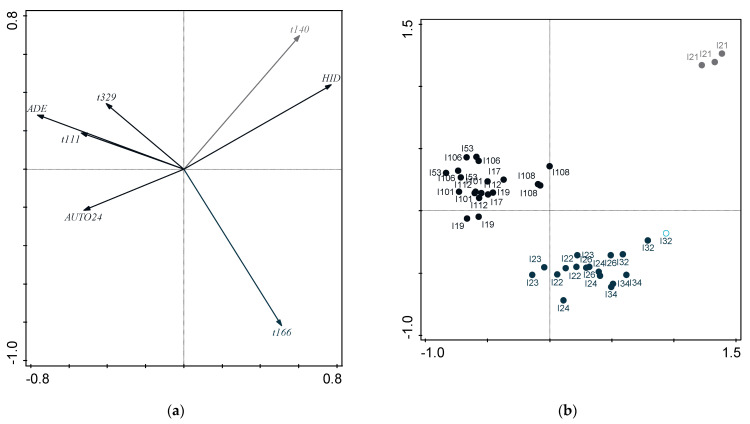
(**a**) Plot of loadings formed by first two principal components from Principal Component Analysis, pertaining to probiotic characteristics associated with gastrointestinal tract and time of processing; and (**b**) plot of scores encompassing strains from *Cobrançosa* LAB table olives.

Figure 1 shows a PCA applied to the functional properties of potential probiotic strains, such as Caco-2 cell adhesion, autoaggregation by 24 h, and hydrophobicity (autoaggregation by 5 h and co-aggregation were not considered, as per previous findings in the similarity among strains). The KMO measure of 0.586 confirmed that sampling size was adequate to apply factor analysis—with variables exhibiting significant correlation to each other, according to Bartlett’s test of sphericity (*p* = 0.041). Two components were accordingly selected from the scree plot (Figure 1) and were able to justify 78% of total variance. Component 1 is highly correlated to autoaggregation (C1: 0.902, C2: 0.084), while component 2 is highly correlated to adhesion (C1: −0.0190; C2: 0.947); hydrophobicity (C1: −0.677; C2: −0.529) appears to correlate negatively with both components. Inspection of Figure 2a and Figure 3a indicates that hydrophobicity appears in opposite direction to adhesion and autoaggregation, respectively. Inspection of Figure 2a shows that adhesion correlates with a major part of RAPD profiles (lep104, lep205a, and lep205b); based on the plots of scores (Figure 2b), the cluster containing i17, i22, i23, i53, and i106, emerged from all three clusters—as expected, in view of their RAPD profiles (see Table 1). Figure 3a indicates that strains with higher adhesion performance can be typically found in the final stages of sweetening (111 d) and brining (329 d); Figure 3b also shows that the cluster formed by i17, i24, i53, i101, i106, and i108, is related to that of processing times (see Table 1).

### 2.2. Health Benefits

The results pertaining to potential health benefits upon the human being, regarding the 14 LAB native strains from *Cobrançosa* and two probiotic reference strains (from animal and plant sources), are depicted in Table 4 and Table 5.

#### 2.2.1. Antibiotic Resistance

All native LAB cultures assessed exhibited resistance to vancomycin, ofloxacin, and streptomycin; and susceptibility to ampicillin, erythromycin, tetracycline, chloramphenicol, and cephalothin (see Table 4). Strain i112 and the yoghurt control strain exhibited intermediate resistance to ofloxacin, yet their inhibition zones were very close to the “Resistant score” (i.e., halo ≤ 14 mm). Therefore, the resistances of our strains were quite similar to those of either reference strain.

#### 2.2.2. Antimicrobial Activity

Every LAB culture presented no significant inhibition halo around the well when neutralized cell-free supernatant was added. However, blurred halos were observed when the acidic CFS (cell-free supernatant) was used.

#### 2.2.3. Antioxidant Activity

Statistical differences between LAB cultures were found at the 5% level of significance (see ANOVA *p*-value in Table 5); results ranged from 18% (strain i23) to 23% (strain i19). Additionally, the antioxidant activities of all our strains (except i22 and i23) were statistically higher (denoted by superscripts ^a^ or ^ab^) than those of the reference strains, viz. 18% and 13% for yoghurt (denoted by superscript ^bc^) and Greek olives (denoted by superscript ^cb^), respectively.

#### 2.2.4. Cholesterol Assimilation

Statistical differences between cholesterol content of LAB cultures were found at the 5% level of significance (see ANOVA *p*-value in Table 5). Strain i23 presented the highest cholesterol assimilation percentage (83%, denoted by superscript ^a^), despite statistical equivalence to all strains except i24, i101, i17, i108, and i32; the lowest assimilation was exhibited by the latter (68%, denoted by superscript ^e^). All LAB strains exhibited significantly higher values than that of the yoghurt reference strain—53% (denoted by superscript ^f^). Compared to the Greek reference strain—77% (denoted by superscript ^abcd^)—all strains presented significantly equivalent percentages of cholesterol assimilation.

#### 2.2.5. Proteolytic Activity

All studied strains showed proteolytic activity, detected by clear zones around the colonies (see Table 5). Such zones were not identical for all strains, yet the variations in diameter were sufficiently small to merit report.

#### 2.2.6. EPS Production

All strains of *L. pentosus*, except i32, tested positive for EPS production—as per the appearance of white colonies; a positive result was found only for *L. paraplantarum* strain i21 (see Table 5).

#### 2.2.7. Enzymatic Activity

As shown in Table 6, all LAB strains exhibited high activities of aminopeptidase (regarding leucine arylamidase and valine arylamidase), acid phosphatase, naphthol-AS-BI-phosphohydrolase, β-galactosidase, and α-glucosidase; β-glucosidase activity was recorded as high for all strains, except the yoghurt reference strain. No strains exhibited lipase, trypsin, α-chymotrypsin, β-glucuronidase, N-acetyl-β-glucosaminidase, α-manosidase, or α-fucosidase activities. All strains, except i21, i17, i22, i23, i34, and i106, presented high esterase and lipase/esterase activities. Regarding the yoghurt reference strain, these two activities were found to be high, while the Greek strain exhibited low activities. Finally, α-galactosidase activity was recorded as low for strains i21, i17, i22, i23, i34, i106, and the Greek strain; and was absent in the other strains. The same strains possessed no alkaline phosphatase activity—similarly to the reference strains, and unlike the remainder (with low activities).

## 3. Discussion

Adhesion performance to Caco-2 cells in vitro—which mimics in vivo epithelial adhesion on the colon—is crucial for a microorganism to exhibit a probiotic effect. Probiotics must anchor in the colon to exert their action and provide an opportunity to interact with local bacteria and the host’s own cells. The results of our strains (from 1% to 23%) are similar to those of Luz et al. [25], with values between 3% and 19%, and of Maragkoudakis et al. [26], ranging from 0.2% to 25.5%; the latter considered adhesion capacity above 13% to be sufficient.

The ability of bacteria to stick to hydrocarbon moieties constrains their extent of adhesion to epithelial cells in the colon, known as cell surface hydrophobicity [27]; the higher the hydrophobicity, the higher the adherence ability [28]. The values of hydrophobicity of our strains in contact with xylene, as model hydrocarbon, varied significantly among them—16–56%; likewise, Abouloifa et al. [17] reported viz. 15–35%. Similar to Benítez-Cabello et al. [29], our study was directed at an opposite relation between hydrophobicity and adhesion to cell lines. This is, probably, a consequence of the fact that attachment to surfaces depends not only on hydrophobicity of the cell surface, but also on Brownian movement, van der Waals attraction, and surface electrostatic charges. Another explanation is the possibility that microorganisms switch between hydrophobic and hydrophilic phenotypes, in response to changes in environmental conditions and growth phases [29]. Motey et al. [30] and Joghataei et al. [31]—who also found a peculiar anticorrelation—claimed that electrostatic power and cell surface charges originating from proteins, glycoproteins, teichoic and lipoteichoic acids, and exopolysaccharides on the cell wall, may in fact be expressed differently from strain to strain [32].

The data of autoaggregation performance by 5 h of incubation, viz. 19–32%, were similar to those of Abouloifa et al. [17], viz. 10–41%; whereas data collected by 24 h of incubation, viz. 59–93%, presented similarities to those of Margalho et al. [4], viz. 69–99%. Reasoning for having tested those two incubation times arises from the experimental conditions selected among similar studies, e.g., 5 h [17,33] or 24 h [34,35]. However, the former appears to not be sufficiently discriminatory between strains. Autoaggregation ability ensures that the probiotic strain reaches a high cell density in the gut, thus contributing to the adhesion mechanism by forming a protective barrier on the intestinal mucosa and epithelial cells, and promoting temporary settlement [4,36].

Co-aggregation of a strain with a pathogen is expected to play an important role in its elimination from the gastrointestinal tract—namely, via formation of a barrier that prevents colonization by pathogenic bacteria, and enhances production of antimicrobial substances [31,37]. Our values for co-aggregation with pathogenic strains ranged from 16 to 45%. These results agree with Luz et al. [25], viz. 9–31% for LAB strains vs. *Escherichia coli*, *Salmonella enterica*, and *Listeria monocytogenes*; with Joghataei et al. [31], viz. 28–37% vs. *E. coli* and 25–51% vs. *Salmonella typhimurium*; and with Peres et al. [18], viz. 10%–50% vs. *E. coli*, *S. aureus*, *S. typhimurium*, *L. monocytogenes*, and *Helicobacter pylori*. Regarding *Candida albicans*, similar results were reported by Jørgensen et al. [38]. Despite consistent results, this test failed to differentiate between strains, given their statistical similarity.

Resistance towards antibiotics associated with probiotic food consumption—together with evidence of horizontal gene transfer from beneficial bacteria to pathogenic bacteria in host’s gut [36]—has become a public health concern; this accordingly justified inclusion of this test in our study. Resistance of our strains to streptomycin and vancomycin, and susceptibility to chloramphenicol, have also been reported by Ozkan et al. [28]; susceptibility to erythromycin, ampicillin, chloramphenicol, and cephalothin, as well as resistance to vancomycin, have also been reported by Abouloifa et al. [17]. Ofloxacin resistance has been claimed by Zarour et al. [39]; Vergalito et al. [40] and Luz et al. [25] have shown susceptibility to tetracycline, as well. Regarding the other antibiotics, our results did not always agree with the literature—probably due to strain-dependent behaviours [40]. Absence of antibiotic resistance has been outlined in FAO/WHO probiotic selection guidelines as a crucial safety parameter, as reported by Ozkan et al. [28]. Transmissible resistance to antibiotics, e.g., to tetracycline encoded by host mobilizable plasmids, would be a threat; however, our strains did not convey this [39]. Although resistance to vancomycin has been recorded, it is known to be intrinsic and due to chromosomal genes, with no expected transfer of antibiotic resistant genes from LAB to pathogens; furthermore, Ozkan et al. [28] showed that resistance to vancomycin and streptomycin is naturally present in LAB strains, rather than acquired through gene transfer. Therefore, the risk of lactobacillemia caused by probiotic *Lactobacillus* has been considered unequivocally negligible [17]. Conversely, probiotic strains with intrinsic resistance to antibiotics may prove useful in restoring intestinal microbiota, following treatment therewith [39].

Antimicrobial activity against pathogens is deemed a desirable trait, although not mandatory for probiotic performance [41]. Our results did not support clear conclusions in this regard, consequently this test was discarded at early stages in the selection process of probiotic strains.

Antioxidant compounds produced by probiotics protect the human body against high levels of oxygen radicals—known to cause damage to lipids, proteins, and DNA—and accordingly help prevent many diseases, e.g., cardiovascular diseases, diabetes, and ulcers in the gastrointestinal tract [17,36]. Compared to data conveyed by Abouloifa et al. [17], viz. 43–93% (via the same method), our results were considerably lower, viz. 16–23%; comparatively, Oliveira et al. [20] reported even lower values, 3–17%. Ayyash et al. [42] claimed antioxidant values of 5–20% on the day of the experiment, depending on the strain; but an increasing trend until 21 days of incubation—when values ranged within 10–50%. Therefore, antioxidant activity may increase with residence time in the gut.

Recent discoveries have also linked probiotic ingestion to prevention of heart disease, by lowering serum levels of cholesterol and decreasing its solubility which reduces uptake in the gut [36]. Our study produced cholesterol assimilation values between 68 and 82%, thus revealing significantly higher percentages for all strains than those of the yoghurt control strain. Our values were higher than those of Vergalito et al. [40], viz. 20–50%, despite the use of similar methodology.

It is important that probiotic strains possess proteolytic activity, as this facilitates nutrient procurement once in the gastrointestinal tract; additionally, bioactive peptides may be produced [18,43]. Although all strains exhibited proteolytic activity, the qualitative data were not conclusive enough to support discrimination between them.

Another criterion for probiotic activity is EPS synthesis owing to secondary metabolites ability to increase quality and shelf life in fermented foods [36,44]; EPS production in our study was observed for most *L. pentosus* strains (except i32), but only one *L. paraplantarum* strain (i21). Abouloifa et al. [17] reported EPS production in *Levilactobacillus* (*Lv.*) *brevis* and *L. plantarum* strains; Ozkan et al. [28] in *Lv. Brevis*, *L. plantarum*, *Lc. Paracasei*, *Loigolactobacillus coryniformis*, *Lc. Rhamnosus*, and *Lactobacillus helveticus*; and Peres et al. [18] in *L. plantarum* and *L. paraplantarum*.

LAB strains presented a broad spectrum of enzymatic activities, yet our results were essentially similar to those in the literature [17,35]. β-glucosidase activity was high for all our strains, except the yoghurt reference strain; this was expected, as this enzyme is responsible for degrading oleuropein, a natural component of table olives. This activity is very important to remove natural fruit bitterness, or reduce the time needed in olive debittering—known to enhance food flavour and accelerate ripening [45,46,47]. Other enzymatic activities can enhance food flavour; some strains showed positive results for esterase and esterase/lipase, which support lipolyzed flavours. Other examples include α-glucosidase activity, which catalyses degradation of saccharose into sweeter fructose via hydrolysis of α-glycosidic linkages of the non-reducing end of oligosaccharides and polysaccharides [35]; and aminopeptidase activity, which plays an important role toward hydrolysis of bitter peptides, and concomitant release of amino acids [17,25]. Additionally, acid phosphatase and naphthol-AS-BI-phosphohydrolase activities were found to be high; in the human digestive system, both are necessary to free phosphoryl groups from other molecules during digestion, aside from improving food maturation [25,47]. Contrarily, some enzymes should be absent in probiotic strains. This is notably the case of N-acetyl-β-glucosaminidase activity, which may raise a safety concern for application in foods, as it releases toxic products, and transforms many pro-mutagens and pro-carcinogens into their mutagenic and carcinogenic forms, respectively [48]; in addition, being associated to the development of several gastrointestinal diseases [40,49]. Furthermore, trypsin, α-chymotrypsin, and β-glucuronidase, are also associated with intestinal diseases [17], thus should be absent in potential probiotic strains. Low activities toward carbon sources such as α-mannose and α-fucosidase, as well as high β-galactosidase and α- and β-glucosidase activities, suggest that our strains have a preference for lactose and glucose as carbon and energy sources [47]. Alkaline phosphatase (responsible for dephosphorylation) and lipase (bearing lipolytic activity) activities were quite low, even absent, in our strains. β-Galactosidase activity was found to be high among our strains and is relevant for its ability to alleviate the symptoms of lactose intolerance following dairy product ingestion, or intolerance to galactooligosaccharides that serve as specific nutrients to probiotics already established in the gut [25].

In attempts to select the best adventitious (potentially) probiotic strain in *Cobrançosa*, analysis should depart from the functional properties of the strains under scrutiny. LAB isolates bearing low values for hydrophobicity exhibited high values for adhesion and autoaggregation, and vice versa. The variable hydrophobicity is thus redundant, consequently it was not considered as a selection criterion.

The best candidates to probiotic LAB strains, based on functional properties that include RAPD profiles, are i17, i22, i23, i53, and i106. Considering Margalho et al. [4] labelled 24 h-aggregation percentages as “high” when above 70%, and “moderate” if between 20% and 70%, our strains i17, i23, i53, and i106, would accordingly rank as high, unlike strain i22 (59%). Therefore, we decided to discard i22 as a best candidate for a probiotic culture tailor-designed in the addition to *Cobrançosa* table olives in a future standardized process.

The best candidates to probiotic LAB strains, based on functional properties including processing time, were strains i17, i24, i53, i101, i106, and i108; this is probably a consequence of ecological dominance of the most resistant strains by the final time of each stage.

Of the two aforementioned analyses regarding functional properties, the isolates selected for further consideration were i17, i53, and i106. This selection is consistent with Table 2, as these isolates exhibited good values for autoaggregation (84, 93, and 89%, respectively); the same did not occur with adhesion (14, 22, and 23%, respectively), for which i17 exhibited significantly lower adhesion performance. Therefore, strain i17 was discarded on statistical grounds, thus leaving only i53 and i106 as statistically identical for both criteria.

The next screening step relied on health benefit features, as per in vitro assessment. Antibiotic resistance and proteolytic activity could not be used as criterion since performance of isolates were very similar to each other (Table 4). The isolates i53 and i106 produced EPS (+), exhibited antioxidant performance (ca. 20%), and cholesterol assimilation (ca. 78.5%), statistically equivalent to each other (see Table 5); hence, no strain was discarded from further analysis based on these criteria. Finally, such isolates revealed positive activity of advantageous enzymes (see Table 6): aminopeptidases, acid phosphatase, naphthol-AS-BI-phosphohydrolase, β-galactosidase, and α- and β-glucosidase; along with negative results pertaining to enzymes associated with safety concerns for human health: trypsin, α-chymotrypsin, β-glucuronidase, and N-acetil-β-glucosaminidase.

Since differences were not substantial between strains, i53 and i106 remained the best candidates as a starter or adjunct culture possessing a high probiotic potential—on average, gathering the best probiotic features. Finally, the addition of strain i106 to *Cobrançosa* table olives had previously been tested in loco (at pilot scale) to ascertain its viability and effects upon the technological, microbiological, and physicochemical profiles along the fermentation process, as well as upon the sensory features thereof. Such experiments were run in parallel with a control using only the spontaneous microflora and supported the conclusion that it is technologically feasible to use those potentially probiotic strains as additives, without compromising the expected quality of the final product [50]. However, addition of that strain is likely to enhance the added value of the final product, for the associated potential benefits upon human health.

## 4. Materials and Methods

### 4.1. Bacterial Strains and Growth Conditions

Fourteen LAB strains belonging to genus *Lactiplantibacillus*, and species *paraplantarum* and *pentosus* were tested in this study (see Table 1); they were previously collected from *Cobrançosa* table olive brines [24]. These strains were identified by combining RAPD-PCR and 16S rRNA sequencing, according to the method described by Reis et al. [24]; 16S rRNA sequences of these strains were deposited in the NCBI GenBank database, under convenient accession numbers (see Table 1) [51].

In our laboratory, they were first screened for technological characteristics (i.e., ability to survive/grow at different salt concentrations, ability to survive/grow at high and low pH, capacity to degrade/assimilate oleuropein, and tendency to produce CO_2_) (unpublished data); food safety features (i.e., mucin degradation ability, and haemolytic and DNase activities); and gastrointestinal survival. Such strains, stored in 15% glycerol at −80 °C, when not in use, were revived in Man, Rogosa, and Sharpe (MRS) agar (VWR Chemicals, Leuven, Belgium), at 30 °C for 48 h, then maintained at 4 °C for upward of one week. Prior to the experiments, they were subcultured in MRS broth (VWR), at 30 °C for 15 h, without shaking (i.e., overnight incubation), to attain the stationary phase. A strain of *Lacticaseibacillus* (*Lc.*) *casei* isolated from a well-known commercial brand of probiotic yoghurt was used as the probiotic reference strain; in addition, a *Lactiplantibacillus* (ex. *Lactobacillus*) *pentosus* strain B281 bearing probiotic properties, previously isolated from Greek table olives, kindly provided by the Laboratory of Microbiology and Biotechnology of Foods, Department of Food Science and Human Nutrition, Agricultural University of Athens [8].

### 4.2. Strain Selection Based on Functional Properties

#### 4.2.1. Caco-2 Cell Adhesion

The method of Argyri et al. [52] was hereby followed. Caco-2 cells (2 × 10^5^) (from European Collection of Cell Cultures, n. 86010202) were seeded in 12-well plates with Dulbecco’s modified Eagle’s medium—composed of 2 mM glutamine, 1% (*w*/*v*) nonessential amino acids, and 20% (*v*/*v*) fetal bovine serum, supplemented with 100 U/mL penicillin/streptomycin (Sigma Aldrich, St. Louis, MO, USA). Medium in the wells was replaced by fresh medium every 2–3 days, and monolayers maintained for 13 days in 7% CO_2_ at 37 °C until lack of further (visible) differentiation. Before proceeding to Caco-2 monolayers, the growth medium in the tissue culture plates was withdrawn, cells were washed twice with 3 mL of Phosphate-Buffered Saline (PBS, i.e., 10 mM Na_2_HPO_4_, 1 mM KH_2_PO_4_, 137 mM NaCl, and 2.7 mM KCl (VWR), pH 7.2), and incubated at 37 °C for 1 h with 2 mL of RPMI-1640 without serum or antibiotics. Before performing the adhesion assay, all bacterial cultures were grown overnight until the stationary phase in MRS at 37 °C, centrifuged for 10 min at 4 °C and 3211× *g* (Model 1580R, GYROZEN, Gimpo, Korea), washed twice with sterile PBS, and finally diluted in RPMI-1640 medium (without serum or antibiotics) to 10^7^ CFU/mL. Subsequently, 1 mL of bacterial RPMI suspension was added to each well (technical replicates), with each strain being assessed for adherence in triplicate for each experiment. Following co-incubation for 2 h at 37 °C under 5% CO_2_/95% air, the medium was removed, and the monolayer washed three times with 1 mL of sterile PBS. The Caco-2 cells were detached with 2 mL of 1% of Triton X-100 (*v*/*v*) (Sigma Aldrich, St. Louis, MO, USA) in PBS. Following incubation for 5 min at 37 °C, cell lysates were serially diluted and plated on MRS agar. The experiments were repeated three times (biological replicates) for all strains. Adherence performance, expressed as percentage, *Ad*(%)*,* was calculated via Equation (1):

(1) $$Ad\left(\%\right)=\left(\frac{CFU_{f}}{CFU_{i}}\right)\times 100$$

where *CFU_f_* = number of bacterial cells that remained attached and *CFU_i_* = total number of bacterial cells initially added to each well.

#### 4.2.2. Hydrophobicity

The hydrophobicity of the strain cell surface was evaluated by measuring cell adhesion to hydrocarbons such as *o*-xylene. As described by Abouloifa et al. [17], overnight cultures were centrifuged (3211× *g*, 10 min, 5 °C), washed twice in sterilized PBS, and re-suspended in 0.1 M sterilized KNO_3_ (VWR) to an optical density at 600 nm, adjusted to 10^8^ CFU/mL. Next, 3 mL of cell suspension was mixed with 1 mL of *o*-xylene (Sigma Aldrich), and the mixture left at room temperature (25 °C) for 10 min. It was then vortexed for 2 and 20 min to promote phase separation. The aqueous phase was collected, and its absorbance recorded at 600 nm. This assay was performed in triplicate for all strains (biological replicates). The cell surface hydrophobicity was expressed as percentage, *H*(%), using Equation (2):

(2) $$H\left(\%\right)=\left(1-\frac{OD_{f}}{OD_{i}}\right)\times 100$$

where *OD_i_* = optical density at start of assay and *OD_f_* = optical density at end of assay.

#### 4.2.3. Autoaggregation

Following Abouloifa et al. [17], LAB cultures were harvested by centrifugation (3211× *g*, 10 min, 5 °C), after overnight incubation in MRS broth at 30 °C. The pellet was washed twice and re-suspended in PBS to obtain 10^8^ CFU/mL. The initial absorbance at 600 nm was measured, and the bacterial suspension was then incubated at 30 °C for 5 h. An aliquot was collected from the surface of the sample, and its absorbance measured at 600 nm; this measurement was repeated by 24 h. All aforementioned experiments were repeated three times (biological replicates) for all strains. Autoaggregation performance was expressed as percentage, *A*(%), according to Equation (3):

(3) $$A\left(\%\right)=\left(1-\frac{OD_{f}}{OD_{i}}\right)\times 100$$

where *OD_i_* = optical density measured at start of assay (time 0) and *OD_f_* = optical density measured at end thereof (by 5 or 24 h).

#### 4.2.4. Co-Aggregation

The methods by Montoro et al. [53] and Taheur et al. [54] were followed. LAB cultures were incubated overnight in MRS broth at 30 °C, without agitation. The pathogenic strains were incubated at 37 °C with agitation, in appropriate media: *Bacillus cereus* ATCC 11778 in Tryptic Soy broth with Yeast extract (TSY, VWR); *Candida albicans* ATCC 10231 in Yeast extract Peptone Dextrose broth (YPD, VWR); *Escherichia coli* ATCC 25922 and *Salmonella enteritidis* ATCC 25928 in Luria Broth (LB, VWR); and *Enterococcus faecalis* ATCC 29212, *Listeria innocua* ATCC 33090, and *Staphylococcus aureus* ATCC 25923 in Brain Heart Infusion (BHI, VWR). All cultures were harvested by centrifugation (3211× *g*, 10 min, 5 °C), washed and re-suspended in PBS, and adjusted to 10^8^ CFU/mL. Then, 2.4 mL of a LAB suspension was mixed to 2.4 mL of pathogen suspension. As controls, 4.8 mL of each LAB strain, and 4.8 mL of each pathogenic strain were used. Absorbance at 600 nm of each bacterial suspension was measured at time 0. By 24 h of incubation at 37 °C, absorbance of the upper suspension was recorded. This assay was performed in triplicate for all strains (biological replicates). Co-aggregation performance, *CA (*%*)*, was calculated via Equation (4):

(4) $$CA\left(\%\right)=\left[{\left(OD_{t}-OD_{t0}\right)}_{mix}-\left(\frac{{\left(OD_{t}-OD_{t0}\right)}_{LAB}+{\left(OD_{t}-OD_{t0}\right)}_{pat}}{{\left(OD_{t0}\right)}_{LAB}+{\left(OD_{t0}\right)}_{pat}}\right)\right]\times 100$$

where *OD_t_*_0_ = optical density measured at start of assay and *OD_t_* = optical density measured at end of assay, for the “*mix*” (of lactic acid bacteria and a pathogenic strain), “*LAB*” (lactic acid bacteria)”, or “*pat*” (pathogenic strain).

### 4.3. Health Benefits

#### 4.3.1. Antibiotic Resistance

Susceptibility to antibiotics was measured by the agar disc diffusion method, using representatives from different groups (e.g., β-lactams, aminoglycosides), and comprising diverse mechanisms of action (e.g., inhibition of cell wall, inhibition of protein synthesis). The final selection was 30 μg/mL of chloramphenicol, 30 μg/mL of tetracycline, 10 μg/mL of ampicillin, 30 μg/mL of cephalothin, 15 μg/mL of erythromycin, 10 μg/mL of streptomycin, 30 μg/mL of vancomycin, and 5 μg/mL of ofloxacin (Oxoid, Thermo Fisher, Basingstoke, UK). Our testing followed Abouloifa et al. [17] and Szutowska et al. [55], with modifications. Lactic acid bacteria revived in MRS agar were inoculated in ca. 3 mL of 0.85% NaCl solution, to obtain a bacterial suspension of 10^5^ CFU/mL. Then, 1 mL of each bacterial suspension was pour-plated on MRS agar, and discs containing the antibiotics were deposited on the plates after 15 min of diffusion; these were incubated at 37 °C for 24/48 h. The results were consubstantiated by diameter of inhibition zone around each antibiotic disc and the strains were categorized based thereon. To ascertain reliability of antibiotic discs used, the same procedure was repeated for *E. coli* and *S. aureus,* pour-plated in Muller-Hinton agar (VWR).

#### 4.3.2. Antimicrobial Activity

The agar well diffusion method of LAB cell-free MRS broth was followed, as per Abouloifa et al. [56] and Mulaw et al. [57]. For this assay, overnight cultures of LAB strains were incubated in MRS broth with 1% (*w*/*v*) glucose (D+; Sigma Aldrich) at 30 °C. The pathogenic strains and growth media were the same as for the co-aggregation ability test, including 37 °C and stirring. Overnight cultures were centrifuged (3211× *g*, 10 min, 5 °C), washed twice in sterilized PBS, and re-suspended in the same buffer. All bacterial pathogenic strains were seeded in Mueller Hinton agar, to an initial optical density of ca. 0.22 at 600 nm. Pathogenic yeast *Candida albicans* was seeded at an initial optical density of ca. 0.4 at 600 nm. Afterwards, each well was loaded with 100 μL of sterile CFS of LAB cultures and incubated at 30 °C for 24 h for bacteria, or 48 h for yeast. To compare the effect of pH on this assay, all LAB cultures CFS were also neutralized with NaOH (5 M; VWR) and pour-plated with pathogens. The qualitative result of this test was reported as inhibition zones of indicator strains around the wells.

#### 4.3.3. Antioxidant Activity

To assess the antioxidant activity, the decrease in concentration of 1,1-diphenyl-2-picrylhydrazyl (DPPH; Sigma Aldrich) radical brought about by LAB strains was selected, following Abouloifa et al. [17]. LAB strains were grown overnight in MRS broth at 30 °C. The bacterial cells were harvested by centrifugation (3211× *g*, 10 min, 5 °C), and washed twice with a sterile solution of 0.9% NaCl; the resulting pellets were adjusted to 10^8^ CFU/mL and re-suspended in the same solution. The cell suspension (0.5 mL) was transferred into a fresh tube, to which 1 mL of methanolic DPPH radical solution (0.1 mM) was also added. The mixture was mixed vigorously and incubated for 30 min at room temperature in the dark. The resulting solution was centrifuged at 9600× *g* (Model SorvallTM LegendTM Micro 17R, Thermo Fisher Scientific, Frankfurt, Germany) for 3 min at 5 °C; and 300 μL of the supernatant was then transferred into 96-well plates, to measure absorbance at 517 nm. The controls included plain sterile 0.9% NaCl and DPPH solution; the blanks contained only ethanol and cells. The experiments were repeated for all strains as three biological replicates, each with two technical replicates. To calculate the antioxidant activity AA(%), the percent reduction in DPPH was determined via Equation (5):

(5) $$\mathrm{AA}\left(\%\right)=\left(\frac{1-OD_{sample}-OD_{blank}}{OD_{control}}\right)\times 100$$

where *OD_sample_* = optical density measurement of samples, *OD_blank_* = optical density measurement of blank (ethanol mixed with bacterial supernatant), and *OD_control_* = optical density measurement of control (DPPH and distilled water solutions) by the end of the assay.

#### 4.3.4. Cholesterol Assimilation

The selection of strains with lowering-cholesterol ability was performed by in vitro tests using water-soluble cholesterol, Cholesterol-PEG 600—polyoxyethylene cholesteryl sebacate (Sigma Aldrich)—and resorted to colorimetry, with a commercial kit for cholesterol quantification (Sigma Aldrich). According to Benítez-Cabello et al. [46], overnight cultures of LAB were centrifuged (3211× *g*, 10 min, 5 °C), washed twice with a 0.85% NaCl solution, and re-suspended in PBS. After 2 h at room temperature (to simulate starvation), 20 μL of suspension was inoculated in 200 μL of MRS broth and supplemented with 3 g/L of oxgall (Sigma Aldrich) and 0.100 g/L of cholesterol. After incubation at 37 °C for 24 h, samples were centrifuged (9600× *g*, 3 min, 5 °C) and pellet was discarded. The cholesterol was quantified according to manufacturer’s instructions, using a standard curve of absorbance at 500 nm vs. cholesterol concentration. MRS and oxgall without cholesterol solution were used as controls. The experiments were repeated for all strains as two biological replicates, each with two technical replicates. After knowing cholesterol concentration, according to said kit, cholesterol assimilation CA(%) was determined via Equation (6):

(6) $$\mathrm{CA}\left(\%\right)=\left(1-\frac{C_{f}}{C_{i}}\right)\times 100$$

where *C_i_* = initial cholesterol concentration and *C_f_* = final cholesterol concentration in the samples.

#### 4.3.5. Proteolytic Activity

The proteolytic activity of isolates was assayed according to Peres et al. [18]. Overnight cultures were spot inoculated on skim milk agar, composed of 5 g/L casein (Sigma Aldrich), 2.5 g/L yeast extract (Merck), 1 g/L glucose (Sigma Aldrich), and 28 g/L skim milk. After 48 h of incubation at 37 °C, the clear zones around the colonies were checked and taken as indicator of proteolytic activity.

#### 4.3.6. Exopolysaccharide (EPS) Production

The ruthenium red staining method, by Abouloifa et al. [17] and Anagnostopoulos et al. [58], was chosen to assess EPS synthesis. Starting with overnight cultures of LAB, 5 μL was deposited on the surface of plates containing ruthenium red milk medium composed of 0.5% yeast extract (Merck), 10% skim milk powder (Sigma Aldrich), 1% sucrose (Sigma Aldrich), 0.08 g/L ruthenium red (Sigma Aldrich), and 15 g/L bacteriological agar (VWR). The plates were incubated at 37 °C for 48 h, then colony colour was checked. Ruthenium red is a carbohydrate-binding dye used to stain biofilms formed by EPS-producing bacteria; as a result, the non-ropy strains should develop red colonies due to bacterial cell wall staining, while EPS strains should appear as white colonies.

#### 4.3.7. Enzymatic Activity

The procedure followed to assay for enzymatic activity included API ZYM system (BioMérieux, Marcy-l’Étoile, France), for its coverage of a wide range of enzymes; the procedure was previously described by Abouloifa et al. [17] and Taheur et al. [54]. The overnight LAB cultures were centrifuged (3211× *g*, 10 min, 5 °C), washed, and re-suspended in 0.85% of NaCl solution, at initial optical density of 0.08 at 600 nm. Each well of API ZYM system was loaded with 65 μL of LAB suspension. After 4 h of incubation at 30 °C, reagents ZYM-A and ZYM-B were added to each well, according to manufacturer’s instructions [47]. The enzymatic activity was evaluated based on APY ZYM system scale, as per the manufacturer; the results were first graded from 0 to 5, depending on colour intensity developed within 5 min vis-à-vis with colour reaction chart; then, the values obtained (0–5) were approximately converted to nanomole of hydrolysed substrate (0–40). Each strain was tested three times with the API ZYM kit, to ensure reproducibility of experimental results.

### 4.4. Statistical Analysis

One-way analysis of variance (ANOVA), followed by Tuckey Post-Hoc, equates to multiple comparisons being performed for epithelial adhesion capacity, autoaggregation and co-aggregation abilities, hydrophobicity degree, antioxidant activity, and cholesterol assimilation tests—with the aid of IBM SPSS 27.0 software (IBM, Armonk, NY, USA), at the 5% level of significance. The same software was used for Principal Component Analysis (PCA) with oblique rotation (direct oblimin), applied to Caco-2 cell adhesion, autoaggregation, and hydrophobicity performance; as additionally, determination of Kaiser-Meyer-Olkin (KMO) measure to check that sample size was adequate for analysis, and Bartlett’s test of sphericity to unfold inter-correlations between variables. Furthermore, Canoco™ V5.0 (Microcomputer Power, Ithaca, NY, USA) was used to produce both plot of loadings and plot of scores from PCA applied to adhesion, autoaggregation and hydrophobicity, LAB RAPD profile, table olive fermentation time, and isolate identification.

## 5. Conclusions

All 14 strains of *Lactiplantibacillus paraplantarum* and *Lactiplantibacillus pentosus* tested, adventitious in *Cobrançosa* table olives, exhibited probiotic potential. Comparison with strains from a commercial brand of yoghurt or strains retrieved from Greek table olives unfolded a better performance of L. pentosus strains i53 and i106 in terms of functional properties. Antibiotic resistances were found to be intrinsic—thus discarding the possibility of (undesired) gene exchange with pathogens. The aforementioned strains were able to produce exopolysaccharides and exhibited antioxidant and cholesterol-decreasing activities—likely to bring health advantages to the host.

Limitations of this work were the assessment of antimicrobial and proteolytic activities—which proved not useful for screening, in view of the statistically similar results obtained; more quantitative analytical methodologies are thus recommended.

In future work, probiotic performance of *L. pentosus* i53 and/or *L. pentosus* i106 endogenous lactic acid bacteria strains should be confirmed by resorting to animal models; their inclusion in a starter culture or adjunct culture should be tested at pilot-scale, aiming at a more standardized commercial process.

## Figures and Tables

**Table 1:** Identification of native strains isolated from *Cobrançosa* table olives.

Species	Isolate ID	Time of Process (d)	Producer ID	Tank ID	RAPD Profile
*L. paraplantarum*	i19	166	A	2	lpa01
	i21	140	H	2	lpa06
	i24	111	A	1	lpa01
	i26	166	A	1	lpa01
	i101	111	A	2	lpa01
	i112	111	A	2	lpa01
*L. pentosus*	i17	111	A	2	lpe205a
	i22	166	A	1	lpe205a
	i23	166	A	1	lpe205a
	i32	166	A	2	lpe308
	i34	166	A	2	lpe308
	i53	329	A	2	lpe104
	i106	329	A	1	lpe205b
	i108	329	A	1	lpe308

**Table 2:** Performance of *L. pentosus* and *L. paraplantarum* strains pertaining to Caco-cell adhesion, hydrophobicity, and autoaggregation.

Isolates	Adhesion (% ± SD) *	Hydrophocity (% ± SD) *	Autoaggregation (% ± SD) *	
			5 h	24 h
*L. paraplantarum*				
i19	11.67 ± 2.57 ^efg^	15.79 ± 1.34 ^de^	32.44 ± 4.03	92.86 ± 8.20 ^ab^
i21	6.20 ± 0.96 ^hi^	55.99 ± 1.91 ^a^	23.71 ± 4.92	79.80 ± 5.50 ^abcd^
i24	5.30 ± 0.75 ^i^	21.58 ± 6.67 ^cde^	25.27 ± 6.36	88.69 ± 6.22 ^abcd^
i26	8.37 ± 0.65 ^ghi^	34.13 ± 6.08 ^bc^	19.02 ± 4.49	92.11 ± 4.95 ^abc^
i101	18.40 ± 1.10 ^bc^	19.34 ± 1.08 ^de^	25.31 ± 5.53	88.11 ± 6.42 ^abcd^
i112	15.80 ± 0.36 ^cd^	19.46 ± 5.99 ^de^	23.21 ± 4.01	90.33 ± 6.44 ^abcd^
*L. pentosus*				
i17	13.83 ± 2.12 ^de^	26.42 ± 4.66 ^bcde^	24.21 ± 11.03	83.57 ± 11.18 ^abcd^
i22	12.20 ± 0.36 ^def^	23.50 ± 3.82 ^cde^	26.73 ± 8.32	59.39 ± 8.15 ^d^
i23	15.97 ± 1.27 ^cd^	21.85 ± 4.91 ^cde^	29.15 ± 6.04	88.87 ± 4.21 ^abcd^
i32	6.00 ± 0.46 ^hi^	40.80 ± 3.76 ^b^	23.67 ± 0.08	78.82 ± 7.98 ^abcd^
i34	1.07 ± 0.38 ^ij^	20.97 ± 7.55 ^cde^	19.63 ± 2.17	81.09 ± 5.40 ^abcd^
i53	22.23 ± 2.21 ^ab^	21.63 ± 7.79 ^cde^	31.36 ± 8.81	92.98 ± 3.04 ^a^
i106	23.03 ± 1.72 ^a^	21.54 ± 1.44 ^cde^	24.91 ± 9.38	88.52 ± 4.47 ^abcd^
i108	4.80 ± 1.50 ^ij^	20.78 ± 3.37 ^de^	22.37 ± 1.09	80.05 ± 4.35 ^abcd^
Reference strains **				
commercial yoghurt	9.86 ± 1.01 ^fgh^	27.39 ± 4.75 ^bcde^	23.51 ± 1.32	78.92 ± 5.25 ^abcd^
Greek olives	21.03 ± 0.15 ^ab^	32.26 ± 4.60 ^bcd^	26.12 ± 1.27	71.66 ± 4.64 ^d^
ANOVA				
*p*-value	0.000	0.000	0.354	0.005

* Values are given as means ± SD of data from replicate experiments. Values in the same column with different superscript letters differ significantly (*p* < 0.05). ** Lacticaseibacillus casei isolated from commercial yoghurt; *Lactiplantibacillus pentosus* B281 isolated from Greek table olives [8].

**Table 3:** Performance of *L. pentosus* and *L. paraplantarum* strains pertaining to co-aggregation with pathogens.

Isolates	Co-Aggregation (% ± SD) *						
	Gram Positive				Gram−Negative		Yeast
	*B. cereus* ATCC 11778	*S. aureus* ATCC 25923	*Ent. faecalis* ATCC 29212	*L. innocua* ATCC 33090	*S. enteritidis* ATCC 25928	*E. coli* ATCC 25922	*C. albicans* ATCC 0231
*L. paraplantarum*							
i19	37.11 ± 8.20	39.24 ± 9.77	41.76 ± 4.36	34.37 ± 4.80	29.94 ± 4.71	29.07 ± 11.45	45.25 ± 8.85
i21	26.67 ± 11.16	31.35 ± 9.95	31.65 ± 5.11	38.00 ± 2.65	22.06 ± 8.83	29.84 ± 10.24	37.02 ± 5.92
i24	29.94 ± 13.72	32.01 ± 10.25	41.80 ± 1.46	39.18 ± 1.57	30.42 ± 7.52	35.22 ± 11.27	42.44 ± 3.99
i26	34.92 ± 14.22	41.62 ± 9.62	39.90 ± 1.33	30.24 ± 4.50	32.63 ± 17.03	31.52 ± 14.19	45.95 ± 9.03
i101	34.33 ± 8.64	34.73 ± 11.39	37.33 ± 5.91	34.81 ± 1.27	34.12± 4.26	33.39 ± 7.22	42.86 ± 5.64
i112	28.58 ± 12.10	34.59 ± 4.77	38.67 ± 5.71	33.82 ± 3.55	32.73 ± 6.57	27.15 ± 8.56	37.49 ± 2.21
*L. pentosus*							
i17	30.67 ± 6.23	22.94 ± 8.23	30.66 ± 6.58	35.03 ± 6.90	21.23 ±9.52	22.56 ±6.07	35.44 ± 3.44
i22	33.27 ± 8.01	37.30 ± 2.71	37.78 ± 6.53	33.29 ± 7.77	24.37 ± 4.86	37.10 ± 10.69	40.24 ± 1.60
i23	31.87 ± 12.65	32.53 ± 15.84	36.84 ± 9.02	40.95 ± 1.25	32.60 ± 13.43	28.35 ± 15.42	40.64 ± 5.01
i32	30.41 ± 8.18	28.86 ± 7.42	45.02 ± 6.41	34.95 ± 2.34	28.14 ± 11.35	28.88 ± 6.48	44.64 ± 6.22
i34	33.66 ± 16.31	39.82 ± 3.91	42.12 ± 4.00	38.34 ± 8.34	35.59 ± 6.66	40.78 ± 4.96	45.01 ± 4.78
i53	36.59 ± 10.00	24.66 ± 2.58	38.26 ± 7.67	39.89 ± 5.78	15.67 ± 5.78	32.82 ± 7.96	44.28 ± 5.63
i106	34.05 ± 6.98	29.45 ± 4.65	38.53 ± 12.50	38.13 ± 0.56	25.27 ± 6.98	28.58 ± 10.90	39.39 ± 2.98
i108	30.03 ± 7.05	34.66 ± 10.54	38.17 ± 1.50	36.75 ± 10.68	24.54 ± 4.63	32.34 ± 5.20	40.94 ± 2.94
Reference strains ^**^							
commercial yoghurt	40.84 ± 8.56	39.26 ± 0.27	36.04 ± 0.39	36.57 ± 4.89	31.04 ± 3.49	35.39 ± 7.08	42.77 ± 4.06
Greek olives	41.13 ± 2.30	36.91 ± 8.56	35.82 ± 8.35	35.90 ± 5.51	35.56 ± 9.13	37.48 ± 6.37	44.93 ± 5.83
ANOVA							
*p*-value	0.932	0.761	0.447	0.658	0.211	0.314	0.373

* Values are given as means ± SD of data from replicate experiments. ** *Lacticaseibacillus casei* isolated from commercial yoghurt; *Lactiplantibacillus pentosus* B281 isolated from Greek table olives [8].

**Table 4:** Performance of *L. pentosus* and *L. paraplantarum* strains pertaining to antibiotic resistance.

Isolates	Antibiotic Resistance (mm) *							
	AMP	E	TE	VA	OFX	C	S	KF
*L. paraplantarum*								
i19	38 (S)	29 (S)	25 (S)	0 (R)	0 (R)	33 (S)	0 (R)	27 (S)
i21	40 (S)	29 (S)	25 (S)	0 (R)	0 (R)	29 (S)	0 (R)	24 (S)
i24	45 (S)	25 (S)	28 (S)	0 (R)	0 (R)	34 (S)	0 (R)	45 (S)
i26	38 (S)	27 (S)	28 (S)	0 (R)	0 (R)	29 (S)	0 (R)	26 (S)
i101	35 (S)	26 (S)	26 (S)	0 (R)	0 (R)	33 (S)	0 (R)	22 (S)
i112	42 (S)	27 (S)	29 (S)	0 (R)	16 (I)	33 (S)	0 (R)	40 (S)
*L. pentosus*								
i17	35 (S)	29 (S)	24 (S)	0 (R)	0 (R)	32 (S)	0 (R)	25 (S)
i22	48 (S)	29 (S)	24 (S)	0 (R)	0 (R)	32 (S)	0 (R)	37 (S)
i23	40 (S)	26 (S)	21 (S)	0 (R)	0 (R)	39 (S)	0 (R)	35 (S)
i32	41 (S)	30 (S)	26 (S)	0 (R)	0 (R)	32 (S)	0 (R)	30 (S)
i34	34 (S)	27 (S)	24 (S)	0 (R)	0 (R)	34 (S)	0 (R)	28 (S)
i53	39 (S)	27 (S)	25 (S)	0 (R)	0 (R)	31 (S)	0 (R)	44 (S)
i106	41 (S)	27 (S)	25 (S)	0 (R)	0 (R)	34 (S)	0 (R)	47 (S)
i108	45 (S)	25 (S)	24 (S)	0 (R)	0 (R)	37 (S)	0 (R)	47 (S)
Reference strains **								
commercial yoghurt	36 (S)	35 (S)	35 (S)	0 (R)	13 (I)	33 (S)	0 (R)	32 (S)
Greek olives	32 (S)	28 (S)	26 (S)	0 (R)	0 (R)	28 (S)	0 (R)	22 (S)

* (R) Resistant: ≤14 mm; (I) Intermediate resistant: 15–19 mm; (S) Susceptible: ≥20 mm; AMP: ampicillin; E: erythromycin; TE: tetracycline; VA: vancomycin; OFX: ofloxacin; C: chloramphenicol; S: streptomycin; KF: cephalothin. ** *Lacticaseibacillus casei* isolated from commercial yoghurt; *Lactiplantibacillus pentosus* B281 isolated from Greek olives [8].

**Table 5:** Performance of *L. pentosus* and *L. paraplantarum* strains pertaining to antioxidant activity, cholesterol assimilation, proteolytic activity, and EPS production.

Isolates	Antioxidant Activity (% ± SD) *	Cholesterol Assimilation (% ± SD) *	Proteolytic Activity **	EPS Production **
*L. paraplantarum*				
i19	23.00 ± 0.89 ^a^	78.64 ± 1.60 ^abc^	+	−
i21	20.10 ± 2.79 ^ab^	75.42 ± 0.67 ^abcd^	+	+
i24	20.20 ± 3.40 ^ab^	70.65 ± 4.37 ^de^	+	−
i26	22.27 ± 2.44 ^ab^	75.72 ± 3.12 ^abcd^	+	−
i101	20.47 ± 1.95 ^ab^	74.35 ± 1.23 ^bcde^	+	−
i112	18.03 ± 2.91 ^abc^	77.34 ± 1.51 ^abcd^	+	−
*L. pentosus*				
i17	20.90 ± 0.56 ^ab^	73.72 ± 4.09 ^bcde^	+	+
i22	18.77 ± 0.06 ^bc^	75.68 ± 2.73 ^abcd^	+	+
i23	17.47 ± 2.14 ^bcd^	81.51 ± 0.09 ^a^	+	+
i32	20.27 ± 1.07 ^ab^	67.87 ± 0.69 ^e^	+	−
i34	21.73 ± 1.17 ^ab^	77.62 ± 1.01 ^abc^	+	+
i53	19.90 ± 0.72 ^ab^	79.83 ± 2.59 ^ab^	+	+
i106	19.60 ± 1.75 ^ab^	77.45 ± 0.54 ^abc^	+	+
i108	20.97 ± 2.35 ^ab^	72.20 ± 0.89 ^cde^	+	+
Reference strains ***				
commercial yoghurt	17.77 ± 1.67 ^bc^	52.27 ± 3.17 ^f^	+	+
Greek olives	13.27 ± 1.65 ^cd^	76.54 ± 1.23 ^abcd^	+	+
ANOVA				
*p*-value	0.000	0.000		

* Values are given as means ± SD of data from triplicate experiments. Values in the same column with different superscript letters differ significantly (*p* < 0.05). ** (+) positive result; (−) negative result. *** Lacticaseibacillus casei isolated from commercial yoghurt; *Lactiplantibacillus pentosus* B281 isolated from Greek olives [8].

**Table 6:** Performance of *L. pentosus* and *L. paraplantarum* strains pertaining to enzymatic activities.

Detected Enzyme *	*L. paraplantarum*						*L. pentosus*								Reference Strains **	
	i19	i21	i24	i26	i101	i111	i17	i22	i23	i32	i34	i53	i106	i108	Commercial Yoghurt	Greek Olives
Alkaline phosphatase	1	2	1	1	1	1	0	0	0	1	0	0	0	1	0	0
Esterase	3	3	3	3	3	3	1	1	1	3	1	2	1	3	4	1
Lipase esterase	3	3	3	3	3	3	1	1	1	3	1	2	1	3	4	1
Lipase	0	0	0	0	0	0	0	0	0	0	0	0	0	0	0	0
Leucine arylamidase	4	5	4	4	4	4	3	3	3	4	3	5	3	5	5	3
Valine arylamidase	4	4	4	4	4	4	4	4	4	4	4	5	4	5	5	4
Cystine arylamidase	0	3	0	0	0	0	0	0	0	0	0	0	0	0	3	0
Trypsin	0	0	0	0	0	0	0	0	0	0	0	0	0	0	0	0
α-chymotrypsin	0	0	0	0	0	0	0	0	0	0	0	0	0	0	0	0
Acid phosphatase	4	5	4	4	4	4	3	3	3	4	3	4	3	4	4	3
Naphthol-AS-BI-phosphohydrolase	4	5	4	4	4	4	4	4	4	4	4	4	4	4	5	4
α-galactosidase	0	2	0	0	0	0	1	1	1	0	1	0	1	0	0	1
β-galactosidase	3	5	3	3	3	3	5	5	5	3	5	4	5	4	4	5
β-glucuronidase	0	0	0	0	0	0	0	0	0	0	0	0	0	0	0	0
α-glucosidase	3	3	3	3	3	3	5	5	5	3	5	5	5	5	5	5
β-glucosidase	5	4	5	5	5	5	3	3	3	5	3	4	3	4	1	4
N-acetil-β-glucosaminidase	0	0	0	0	0	0	0	0	0	0	0	0	0	0	0	0
α-manosidase	0	0	0	0	0	0	0	0	0	0	0	0	0	0	0	0
α-fucosidase	0	0	0	0	0	0	0	0	0	0	0	0	0	0	0	0

* Scale of API-ZYM^®^ (bioMÉRIEUX, Craponne, France) test used for enzyme quantification: 0 corresponding to “no activity”; 1 corresponding to 5 nmol substrate metabolized and 2 to 10 nmol: “low activity”; 3 corresponding to 20 nmol, 4 to 30 nmol, and 5 to 40 nmol: “high activity”. ** *Lacticaseibacillus casei* isolated from commercial yoghurt; *Lactiplantibacillus pentosus* B281 isolated from Greek olives [8].

## Data Availability

The data presented in this study are available upon request from the corresponding author. The data are not publicly available due to generation thereof from samples from a commercial manufacturer; the original data and their matching to batches, accordingly, requires prior discussion with company CEO prior to topical release.
